# A case of shiitake dermatitis in the United States

**DOI:** 10.1093/omcr/omab071

**Published:** 2021-08-13

**Authors:** Jesus Gomez, Krishna Sharma, Albert Huho, Neal Gregory

**Affiliations:** 1 Upstate Dermatology; Clinical and MOH’s services, 1547 Columbia Turnpike, Castleton on Hudson, NY 12033, USA; 2 Georgetown University, Class of 2024, 3700 O St NW, Washington, DC 20057, USA

**Keywords:** Flagellate dermatitis, shiitake, mushroom

## Abstract

A 65-year-old male presented to our dermatology clinic with a two-day intensely pruritic rash covering his back. The lesions were predominantly on his chest, upper extremity, and back. He denied any prior history of similar rashes and his past medical history was non-contributory. A detailed exposure history revealed the patient had eaten Shiitake mushrooms for dinner 48 hours previously.

Physical examination showed a truncal dominant rash. Close-up examination confirmed the papulovesicular nature of the rash with multiple small vesicles grouped both along the breadth and length of each linear streak on an erythematous background. Biopsies showed spongiosis with micro-vesiculation. Blood work showed a nominal CBC and CRP/ESR and serum IgE. The patient was put on topical steroid and the rash resolved in one week.

With increasing mushroom consumption [1], cognizance of this etiology avoids a diagnostic ‘odyssey’ and prevents recurrence of this very characteristic rash.

## INTRODUCTION

Shiitake dermatitis is a striking rash, more common in Southeastern Asian countries where shiitake mushrooms are eaten commonly. Mushroom consumption in general has increased worldwide [1] in recent years and recognition of this dermatitis in the primary care setting has become more important even in the western setting.

## CASE REPORT

**Figure 1:**
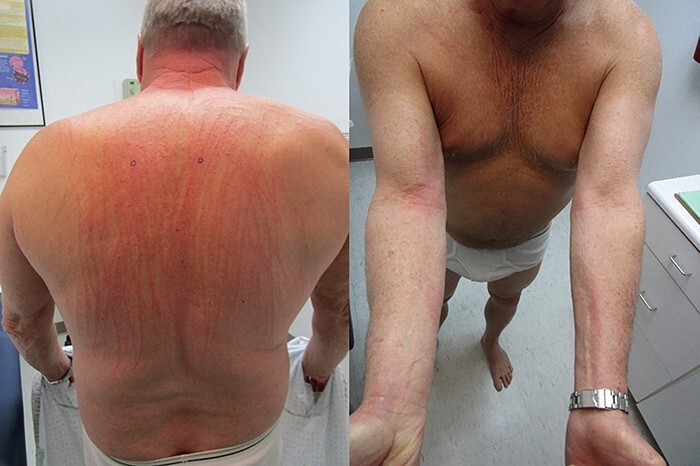
Patient with very characteristic linear rash involving his back(left), neck and antecubital fossa (right).

A 65-year-old retired male (Fitzpatrick phototype II) previously healthy except for a distant history of basal cell carcinoma presented to our dermatology clinic with a two-day duration of a rash. The lesions were first noticed on his chest, upper extremity and back **[**Figure 1**].** The rash was extremely pruritic resulting in insomnia for the previous 2 nights which went unrelieved despite oral diphenhydramine. Also concerning to him and his wife was the whip like nature of the rash which was somewhat linear and run vertically right down his back in a manner unlikely to be produced by simple excoriation. In addition, he denied reaching for or scratching his back in any manner. Review of systems was negative, and he denied any recent history of travel, similarly sick contacts or recent outdoor contactants or change in body soaps or laundry detergents.

He denied any prior history of similar rashes and his past medical history was non-contributory. A detailed exposure history revealed the patient had eaten mushrooms 48 hours ago during dinner at a Chinese cuisine buffet. He admitted to ingesting great quantities of a medley of cooked and raw mushroom including the Shiitake mushroom variety.

**Figure 2:**
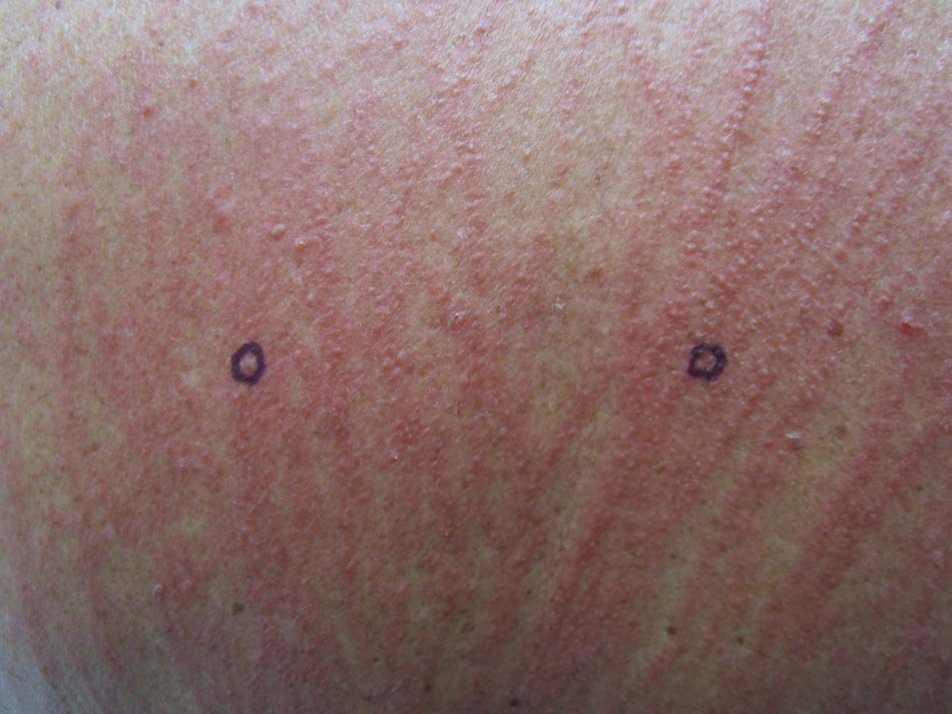
Close up view from the back revealing an erythematous papulovesicular rash. Note multiple papules along the streaks, both longitudinally and side to side along the breadth.

Physical examination was striking with a truncal dominant but generalized rash in a centripetal distribution (Figure 1). The lesions consisted of raised, relatively uniform erythematous papulovesicles arranged in linear arrangement. Close-up views **[**Figure 2**]** confirmed the papulo-vesicular nature of the rash with multiple small papules grouped both along the breadth and length of each linear streak within an erythematous background.

Given the clinical history and presentation the diagnosis was consistent with a shiitake dermatitis. Flagellate patterns of eruption have also been noted in drug reactions particularly to bleomycin, peplomycin (a bleomycin derivative), docetaxel, adult-onset Still’s disease, and dermatomyositis [2]**.**

The patient was put on a mid-potency topical steroid (Triamcinolone 0.1% ointment applied to affected areas twice daily). The rash resolved within 1 week. It has been reported that shiitake dermatitis can also resolve spontaneously.

## DISCUSSION

Shiitake dermatitis is a unique condition that is primarily characterized by the whiplash or flagellate patterned eruptions that it leaves on the extremities and trunks of patients. The first report of this dermatitis was in 1977 in Japan. Nakamura had observed that the ingestion of uncooked or under cooked shiitake mushrooms (*Lentinula edodes*) was causing these peculiar skin eruptions [3]**.** There have since been many more cases reported not only in Asia, but in Europe and the Western Hemisphere as well. It is expected that as the popularity of shiitake mushrooms continues to grow there will be more cases of shiitake dermatitis reported.

There is little known about underlaying pathophysiology. In many cases reported there is often a delay of 24–72 hours between the consumption of the mushrooms and when the eruptions become noticeable, but the possibility of seeing eruptions sooner than that has been reported. In one case reported eruptions started only 5 hours after consuming raw shiitake mushrooms [4]**.** Lentinan, a polysaccharide that is found in shiitake mushrooms is believed to play a role in causing the eruptions [5]**.** Whether it is a toxic reaction or a late hypersensitivity reaction, once ingested the polysaccharide will elicit the secretion of interleukin-1 [6]**.** This cytokine promotes an inflammatory response in the body that results in the whiplash or flagellate patterned eruptions. Heat favors this theory because when fully cooked shiitake mushrooms are consumed no dermatitis is visible. The heat that is used when cooking the mushrooms denatures the lentinan making it nonfunctional.

Bleomycin a drug used in many cancer therapies also could induce flagellate dermatitis [7]. Clinically it may appear that bleomycin and shiitake mushrooms cause the same cutaneous effects, but a major distinguishing characteristic that is seen in bleomycin induced flagellate dermatitis is the visible hyperpigmentation that occurs at the site of the eruptions.

Typically, treatment options for shiitake dermatitis also known as flagellate dermatitis are similar; clinicians can prescribe oral antihistamines and/or topical corticosteroids to help reduce the inflammatory response. As to the duration of the cutaneous eruptions when the dermatitis is caused by the consumption of shiitake mushrooms, most eruptions will clear up within days. Simple avoidance is also important in the prevention of further eruptions. With regards to bleomycin in most cases patients must change their cancer therapy regimen or wait until their regimen is completed before the eruptions begin to resolve. Even after completion it can take several weeks to months for the eruptions to clear up.

## References

[1] Mushroom Market Size, Share & Trends Analysis Report and Segment Forecasts, 2021 – 2028. GRAND VIEW RESEARCH https://www.grandviewresearch.com/industry-analysis/mushroom-market.

[2] Zhao CY. Flagellate erythema: from diet, drugs to dermatomyositis. Med J Aust 2020;213:354. 10.5694/mja2.50784. 32946593

[3] Nakamura T. Shiitake (Lentinus edodes) dermatitis. Contact Dermatitis. 1992;27:65–70. 1395630 10.1111/j.1600-0536.1992.tb05211.x

[4] Adriano AR, Quiroz CD, Acosta ML, Talarico SR, Azulay DR. Shiitake dermatitis: the first case reported in Brazil. An Bras Dermatol. 2013;88:417–9. 10.1590/abd1806-4841.20131849. 23793190 PMC3754375

[5] Hanada K, Hashimoto I. Flagellate mushroom (Shiitake) dermatitis and photosensitivity. Dermatology 1998;197:255–7. 10.1159/000018007. 9812031

[6] Dolly VR, Carlos S. Flagellate dermatitis caused by the intake of shiitake mushrooms. A case report and review of the literature. Rev Alerg Mex 2020;67:79–82. 10.29262/ram.v67i1.620. 32447870

[7] Rachel G, João PN, Marien SS, Marcia R. Bleomycin-induced flagellate dermatitis. BMJ case reports 2013;2013:bcr2013009764. 10.1136/bcr-2013-009764. PMC3702947 23814202

